# Adult-Onset Acute Disseminated Encephalomyelitis Refractory to Steroids

**DOI:** 10.7759/cureus.18669

**Published:** 2021-10-11

**Authors:** Abdulla J AlZeera, Aysha Alkhaja, Dana H Noor, Sayed Mohamed R Alsaffar

**Affiliations:** 1 Internal Medicine, Royal College of Surgeons in Ireland - Medical University of Bahrain, Busaiteen, BHR; 2 Family Medicine, Dr. Jamal Al-Zeera Medical Center, Isa Town, BHR; 3 Neurology, Salmaniya Medical Complex, Manama, BHR; 4 Medicine, King Hamad University Hospital, Busaiteen, BHR; 5 Medicine, Royal College of Surgeons in Ireland - Medical University of Bahrain, Busaiteen, BHR; 6 Consultant Neurologist, Salmaniya Medical Complex, Manama, BHR; 7 Neurology, Ibn Hayan Medical Center, Manama, BHR

**Keywords:** neuroimaging, demyelination, adult-onset, central nervous system, acute disseminated encephalomyelitis (adem)

## Abstract

Acute disseminated encephalomyelitis (ADEM) is an immune-mediated phenomenon characterized by demyelination of the central nervous system that displays numerous manifestations. It predominantly presents in children with a mean age range of five to eight years. ADEM remains a diagnosis of exclusion based on clinical and radiographic progression. Thus, it poses a diagnostic challenge. ADEM has been shown to be very responsive to steroids and exchange therapy.

Here, we present an unusual case of ADEM in a 50-year-old female patient who, despite receiving an entire course of IV methylprednisolone and other conventional treatment methods, did not respond to the treatment.

## Introduction

Acute disseminated encephalomyelitis (ADEM) is a rare, monophasic encephalomyelitis that presents similar to a variety of neurological disorders and thus is difficult to diagnose [1]. It manifests among children in their first or second decade of life. Each year, about one in 125,000-250,000 people is suffering from ADEM. Males tend to be affected more than females (in 1.3:1 ratio) [2].

Progression is rapid as inflammation is directed at myelin and infiltration of vessel wall causing congestion and increased permeability by immune cells. Although multiple etiologies have been proposed, most cases present following acute infections or post-vaccination [2,3]. ADEM is very responsive to steroids; given its immune-mediated pathogenesis, other treatment modalities such as intravenous immunoglobulin (IVIG) and plasma exchange are considered second-line therapies when patients do not take steroids [4].

We describe a case of adult-onset ADEM in a 50-year-old female patient demonstrating clinical deterioration while being treated with high-dose corticosteroids as well as IVIG.

## Case presentation

A 50-year-old female patient presented to the emergency department with diplopia and headache. Three weeks prior to her attendance, she complained of constant back pain that did not improve with conservative treatment. This was her first time experiencing such symptoms. She had no recent trauma or significant changes in her daily routine or diet.

Medical history was remarkable for type 1 diabetes mellites that was consistently controlled with insulin. No other medications were included. Upon medical examination, she was febrile with a temperature of 38.3°C. Ptosis was evident in her left eyelid; a downward and outward ocular positioning consistent with third-nerve palsy was observed with sparing of the pupil. Heart rate, blood pressure, and oxygen saturation on room air were within normal limits. She was fully conscious and oriented to person and time, 5/5 power in all limbs, normal finger-nose test, equivocal plantar reflex as well as negative meningeal signs.

Lab results revealed normal Hb of 14.2 mg/dl, WBC of 13.30 (x 10^9^/L) with 13.9% monocytes, 69.6% neutrophils, 15.9% lymphocytes, 0.1% eosinophils, 0.1% basophils, and C-reactive protein (CRP) of 21 mg/L. All other parameters were within a normal range including urinalysis and liver functions tests. Lab serology for hepatitis C viruses (HCV), HIV, hepatitis B surface (HBS) antigen, and acid-fast bacillus (AFB) smear was all negative. Venereal disease research laboratory (VDRL) screening was also carried out to exclude syphilis. Blood cultures came back negative, and the peripheral blood smear was unremarkable. A lumbar puncture was performed in which cerebrospinal fluid (CSF) analysis revealed a WBC of 175 cells/μL (90% lymphocytes), glucose 7.8 mmol/L, protein 725 mg/L, and a negative AFB smear. Intravenous empiric antibiotics such as Vancomycin, Ceftriaxone, Acyclovir, and Omeprazole were commenced.

**Figure 1:**
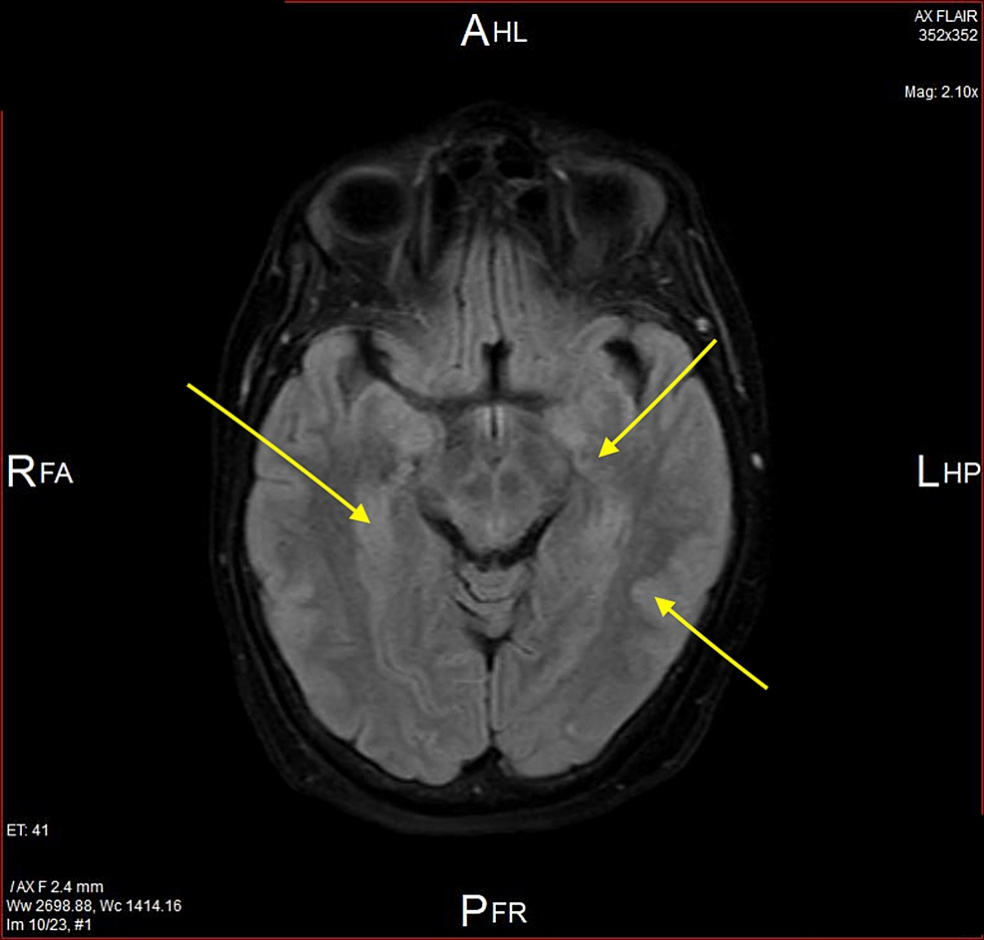
Initial magnetic resonance image (MRI) showing multifocal patchy T2/FLAIR signal abnormalities Yellow arrows in the image indicate multiple hypodense regions. FLAIR, Fluid-attenuated inversion recovery.

On day 2, an MRI was done, which displayed multifocal, patchy-altered signal intensity with lesions involving predominantly the right caudate nucleus, basal ganglia, thalamus, midbrain, interpeduncular cistern as well as tiny foci in dorsal pons. Similar lesions were seen in the right frontoparietal cortex extending along the sulcus. Lesions appeared to be more confluent and prominent on the right side at the basal ganglia region, hypothalamus, and basal cistern. Patchy T2-weighted/fluid-attenuated inversion recovery (T2/FLAIR) hyperintensities are seen in the medulla left side and cervicomedullary junction. Prominent leptomeningeal enhancements were noted along the fourth ventricle, medulla, and upper cervical cord (Figure 1).

No significant lesions were present in the cerebral hemispheres and no evidence of restricted diffusion nor hemorrhage; ventricle cisterns appeared normal with no shifting of the midline structures.

**Figure 2:**
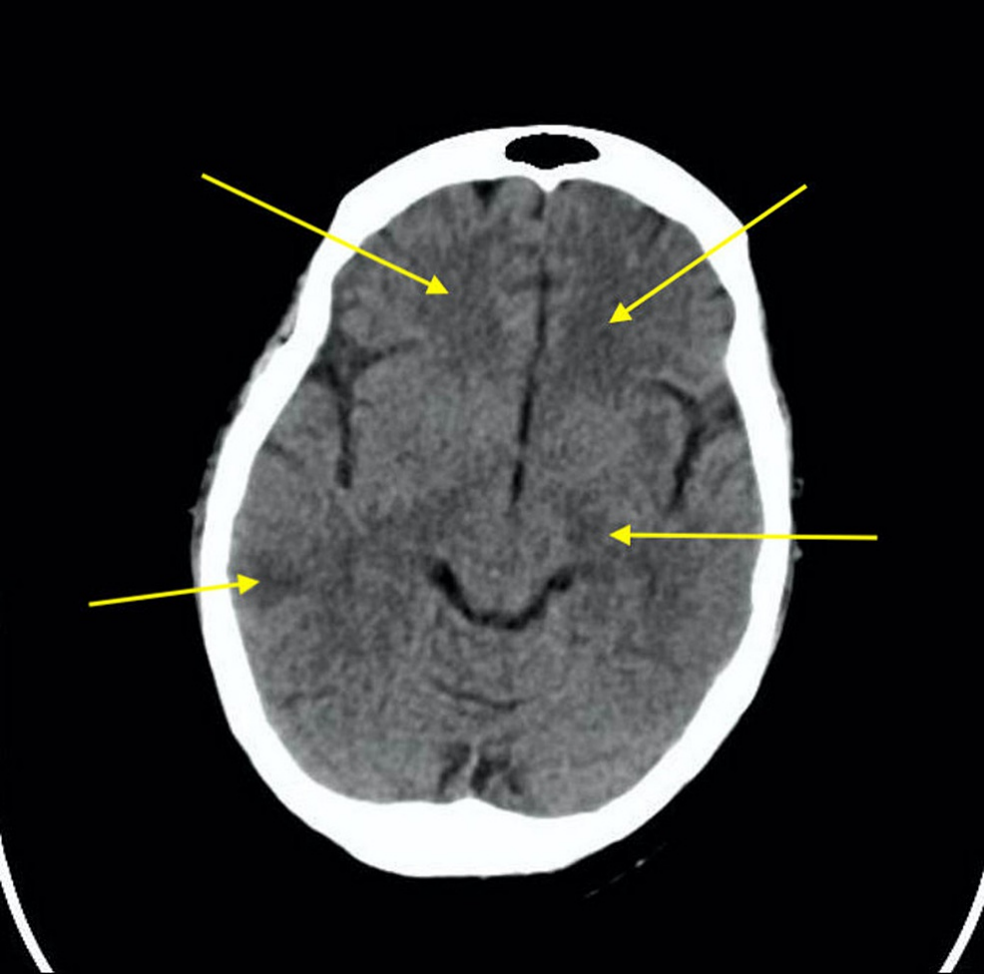
Non-contrast CT of the brain showing axial cuts at the basal ganglia Yellow arrows display the hypodensities in the right parietal lobe, supratentorial, and infratentorial regions.

Suspicion was raised toward ADEM, and the patient was started on IV methylprednisolone for a period of five days while antibiotics and antivirals were discontinued. On day 3, the patient underwent intubation following her transfer to the ICU due to a low Glasgow Coma Scale (GCS) of 9/15 with frothy secretions, tachypnea, and tachycardia. Brain CT was performed, which showed a new large temporoparietal hypodensity in the right side with few supra and infratentorial hypodensities (Figure 2).

The progression of ADEM was observed and suggested that the patient requires continuous monitoring. The patient was on a nasogastric tube and given an osmolyte. A lumbar puncture was done on the same day; CSF analysis showed WBC of 75 cells/μL (80% lymphocytes), glucose of 7 mmol/L, protein of 1573 mg/L, and a negative AFB smear.

**Figure 3:**
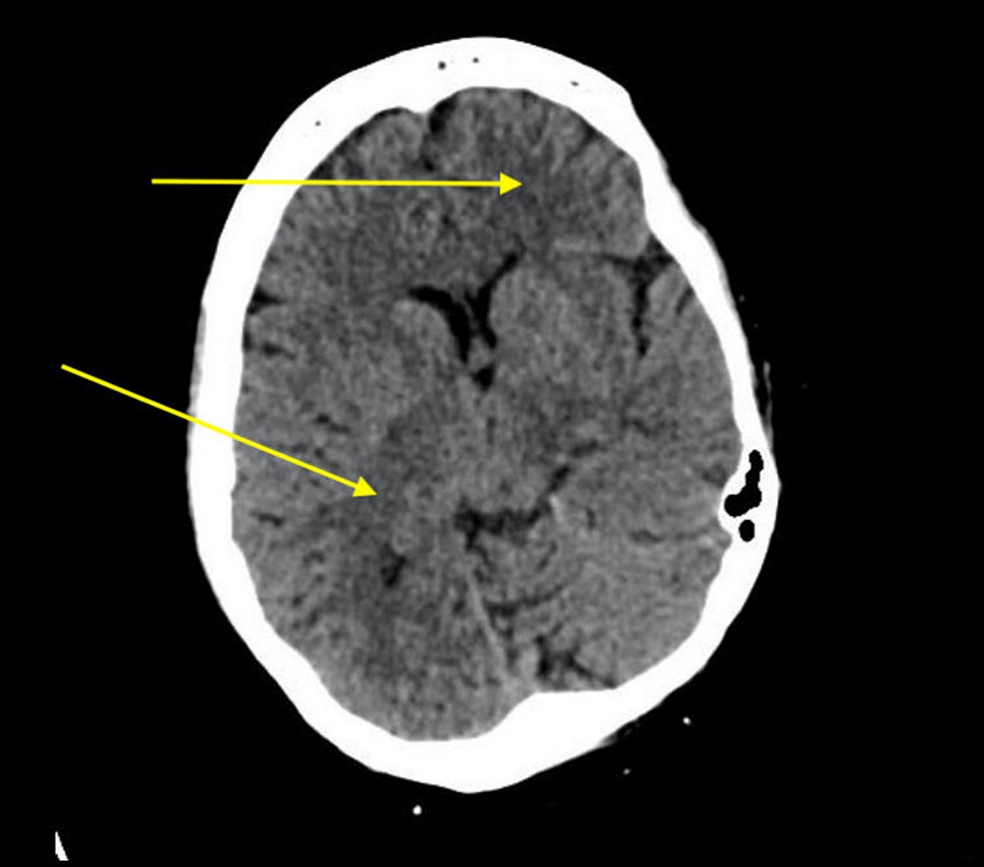
Final non-contrast CT of the brain Yellow arrows show the labeled new hypodensity regions in the frontal lobe and white matter hypodensities in the right high parietal lobe.

On day 4, the patient was not responding to the corticosteroid therapy as clinical deterioration was seen. Pupils appeared unequal during the physical examination; upon non-contrast CT imaging, new hypodensity regions appeared in the frontal lobe in addition to white matter hypodensities in the right high parietal lobe (Figure 3).

As methylprednisolone showed no clinical effect, plasmapheresis was started. Following the treatment regimen, which still showed deterioration on day 5, the patient was started on anti-TB (anti-tubercular) medications. As clinical decline progressed, despite alternative therapies and constant radiological follow-ups, the patient passed away on day 7 due to cardiovascular instability in conjunction with brainstem death as demyelination advanced in numerous areas in the brain.

The case presented here points to an infective etiology backed up by blood tests and CSF findings. Diagnostic imaging with MRI-T2-weighted images demonstrated asymmetrical, bilateral patchy lesions involving multiple brain regions. Multiple sclerosis (MS) was ruled out due to the absence of oligoclonal bands on CSF, the lack of time and space differentiation of brain lesions, and the absence of a corroborating history of recurrence.

## Discussion

ADEM is a rare autoimmune disease that mostly affects children more often than adults by displaying its effects predominantly on the white matter of the brain and spinal cord. The cornerstones of ADEM diagnosis are neuroimaging and laboratory studies alongside the exclusion of similar presentations of multiple diseases such as MS. High-dose intravenous corticosteroids are considered as first-line therapies, given ADEM's responsiveness toward such therapies. IVIGs are the most common alternative therapy for steroid-resistant ADEM.

Although multiple etiologies have been proposed, most cases present following acute infections or post-vaccination [4,5]. Pathogenesis is still unclear, yet it is widely thought to be post-viral (coxsackievirus, cytomegalovirus, Epstein-Barr virus, coronavirus, herpes simplex virus, measles, rubella virus, varicella-zoster virus, and hepatitis A virus) and viral post-vaccination (post-measles ADEM in 1:1000, post-varicella ADEM in 1:10,000, and post-rubella ADEM in 1:20,000) [5,6]. In addition, patient genetics, history of immunization, and exposure to infectious agents (viral or otherwise) have been postulated as potential risk factors for ADEM [2].

On clinical grounds, MRI is a remarkably sensitive diagnostic technique for diagnosing ADEM with a projected 81% sensitivity and a 95% specificity in diagnosing and differentiating ADEM from MS in children [7]. This is important because other demyelinating illnesses, specifically MS, must be ruled out using supporting biological and clinical markers before a diagnosis of ADEM can be reached.

Disease progression is rapid in most instances, with a reported mortality rate of 1%-3% [8,9]. However, it is essential to note a few prognostic indicators that have been associated with a poor outcome. These include fever at admission, ventilator-associated pneumonia, altered sensorium, meningeal irritation, and lower motor neuron involvement [10]. Further, factors such as a history of optic neuritis, familial history of central nervous system inflammatory demyelination, and the lack of neurological sequelae have been associated with an increased risk of ADEM relapse [11]. Overall, though an interesting study showed that an estimate of 80% of pediatric patients experienced complete recovery when treated with high-dose IV methylprednisolone with rapid recovery, others experienced mild neurological deficits [8,12]. However, such data is virtually non-existent in the adult or elderly population and thus can not be extrapolated for our patient.

Based on empirical evidence and observational studies, a pathogenesis-oriented regimen is a preferred method; high-dose corticosteroids are widely accepted as first-line therapy and have demonstrated remarkable outcomes by alleviating central nervous system (CNS) attacks and inflammatory reactions while achieving neurological improvement. Given its immune-mediated pathogenesis, ADEM is very responsive to steroids and alternative treatment modalities such as IVIG and plasma exchange [13]. It should be noted that the role of corticosteroids in patients presenting late in the course of the disease is questionable, as was the case in this patient [13]. When corticosteroids failed to work satisfactorily or were contraindicated, IVIG and plasmapheresis should be examined as alternatives. Several case studies have proved beneficial outcomes and the efficacy of plasma exchange/plasmapheresis in severe attacks of ADEM [14,15].

There was a delay in starting IV methylprednisolone in this instance, and the patient remained unresponsive to the treatment with clinical and neuroradiological progression. Subsequently, plasma exchange was commenced; however, the patient developed cardiac and respiratory complications as the lesions progressed to the brainstem, leading to death.

## Conclusions

ADEM is a demyelinating disorder of the central nervous system and spinal cord, which is described as monophasic with an acute and rapid time of onset. ADEM mainly manifests in the pediatric age group, most often preceded by immunization or an infection with a viral etiology. Due to the lack of distinctive biomarkers and clinical features, the ADEM diagnosis can only be made when other autoimmune diseases that mimic ADEM, such as MS, are ruled out.

Due to its autoimmune nature, the approach to treatment consists of early therapy with steroids. Favorable outcomes have been reported with IV methylprednisolone and consequently IVIG and plasma exchange. Steroid-resistant ADEM is rare, especially in the older age group; however, further research is warranted regarding adult-onset ADEM. Cyclophosphamide, in particular, has shown to be of great benefit in other autoimmune conditions and should be evaluated for ADEM in adults.
